# Changes in ethylene and sugar metabolism regulate flavonoid composition in climacteric and non-climacteric plums during postharvest storage

**DOI:** 10.1016/j.fochms.2022.100075

**Published:** 2022-01-21

**Authors:** Macarena Farcuh, Hiromi Tajima, Larry A. Lerno, Eduardo Blumwald

**Affiliations:** a Dept of Plant Science and Landscape Architecture, University of Maryland, College Park, MD 20742, USA; b Dept of Plant Sciences, University of California, Davis, CA 95616, USA; c Food Safety and Measurement Facility, University of California, Davis, CA 95616, USA

**Keywords:** Japanese plums, Flavonoids, Ethylene, Sugars, Postharvest ripening, RP-HPLC

## Abstract

Plums are rich in flavonoids, key contributors to fruit coloration and putative health benefits. We studied the impact of changes in ethylene and sugars in flavonoid metabolism-related pathways of the climacteric Santa Rosa and its non-climacteric mutant Sweet Miriam, throughout the postharvest period. Fruits were harvested at optimal maturity, subjected to ethylene treatments, and evaluated during storage. We examined transcript profiles of structural and regulatory genes of flavonoid-related pathways and their associated metabolites in skin and flesh, integrated with multivariate analyses of ethylene and sugar metabolism. Ethylene treatments were positively correlated with anthocyanin and negatively correlated with flavonol and flavan-3-ol metabolism. Sucrose and galactose were positively associated with anthocyanin concentration, while sorbitol, fructose, glucose and minor sugars were correlated with flavonol and flavan-3-ol metabolism. Our results support the notion that ethylene is playing key roles in shifting plum fruit flavonoid profiles, which are also associated with changes in fruit sugars.

## Highlights

• Ethylene metabolism regulated flavonoid (and sugar) contents and composition.
• Ethylene induced anthocyanin and reduced flavonol and flavan-3-ols in plum fruit.
• Anthocyanins positively correlate with sucrose and galactose metabolic pathways.
• Flavonol and flavan-3-ols associated with sorbitol, fructose, and glucose contents.

## 1 Introduction

Japanese plums (*Prunus salicina* Lindl.), which include most of the fresh-market plums commercialized worldwide, are rich in flavonoids such as anthocyanins, flavonols and flavan-3-ols (Xu, Huan, Jiang, Zheng, & Brecht, 2020). Anthocyanins contribute to fruit skin and flesh coloration, key for quality and marketability, and together with flavonols and flavan-3-ols protect fruit from photooxidative damage, act as defense agents, and help prevent coronary disease and cancer by eliminating free radicals (Rupasinghe, 2020). Generally, anthocyanins and flavonols are more abundant in skin than flesh tissues, while flavan-3-ols contents are similar in both (Tomás-Barberán et al., 2001, Treutter, 2001).

Flavonoids share common precursors of the phenylpropanoid and flavonoid pathways for their synthesis (Supplementary Fig. S1). Some key enzymes involved in flavonoid biosynthesis include phenylalanine ammonia-lyase (PAL), cinnamate-4-hydroxylase (C4H), chalcone synthase (CHS), chalcone isomerase (CHI), flavanone 3-hydroxylase (F3H), dihydroflavonol 4-reductase (DFR), leucoanthocyanidin dioxygenase (LDOX), and UDP glucose-flavonoid 3-O-glucosyltransferase (UFGT). F3H produces dihydroflavonols, that can be either converted to flavonols via flavonol synthase (FLS) or to leucoanthocyanidin via DFR. Leucoanthocyanidins can then be reduced to flavan-3-ols (catechins) via leucoanthocyanidin reductase (LAR) or dehydrated by LDOX to form anthocyanidins. Finally, anthocyanidins can either be reduced by anthocyanidin reductase (ANR) to form flavan-3-ols (epicatechins) or converted by UFGT to anthocyanins (Zhang et al., 2018). Furthermore, transcription factors such as R2R3-MYB, basic helix-loop-helix (bHLH3) and WD40 proteins have been reported to form a MBW complex that binds to promoters and activate transcription of structural genes of the anthocyanin biosynthesis pathway (Jaakola, 2013). In plums, MYB10 transcript levels have been shown to be upregulated during anthocyanin accumulation (Cheng, Liu, Yuan, & Guan, 2016).

Flavonoid biosynthesis can be affected by light, temperature, hormones and sugars (Huang et al., 2019, Xu et al., 2020, Zhang et al., 2018, Zheng et al., 2009, Zhou et al., 2020). Ethylene has been reported to particularly affect anthocyanin accumulation, yet its effect(s) on other flavonoids is not clear. In climacteric fruits, treatments with 1-methylcyclopropene (1-MCP), an inhibitor of ethylene binding to its receptors (Watkins, 2006) decreased expression of anthocyanin-biosynthesis related genes and anthocyanin contents (Cheng et al., 2016, MacLean et al., 2007, Manganaris et al., 2008, Xu et al., 2020). In non-climacteric fruits, ethylene treatments increased anthocyanin biosynthesis (Chervin et al., 2009, El-Kereamy et al., 2003, Villarreal et al., 2010). Previously we reported that both climacteric and non-climacteric plums showed changes in gene expression associated with ethylene biosynthesis, perception and signaling during postharvest ripening and in response to ethylene treatments (Farcuh et al., 2019), however its relation to changes in flavonoids is poorly understood.

In addition to playing key roles in energy metabolism and contributing to fruit taste (Farcuh, Li, Rivero, Shlizerman, Sadka, & Blumwald, 2017), sugars also act as signaling molecules with a hormone-like signaling function (Duran-Soria, Pott, Osorio, & Vallarino, 2020). Furthermore, the addition of sugar moieties through glycosylation is required for anthocyanin and flavonol synthesis in fruit (Jaakola, 2013). We previously showed the reprogramming of sugar metabolism-related pathways in fruit with contrasting ripening behaviors (Farcuh et al., 2017) and that ethylene treatments during fruit storage differentially affected sugar metabolism of climacteric and non-climacteric fruit (Farcuh, Rivero, Sadka, & Blumwald, 2018). Whether these differences in sugar metabolism-related pathways between fruit with contrasting ripening behaviors are associated with changes in flavonoid-associated metabolism remains to be studied.

We hypothesize that changes in ethylene and sugar metabolism-associated pathways play a key role in the regulation of fruit flavonoid metabolism during postharvest ripening. We examined the expression profiles of structural and regulatory genes of the phenylpropanoid and flavonoid pathways and their associated metabolites, in skin and flesh tissues of the climacteric plum Santa Rosa and its non-climacteric mutant Sweet Miriam fruits treated with ethylene during postharvest storage. Our results support the notion that anthocyanin biosynthesis competes for substrates with flavonols and flavan-3-ols and that changes in ethylene and changes in sugars regulate these pathways. This work could be applied for the identification and manipulation of targets for improvement of plum fruit coloration and health properties.

## 2 Materials and methods

### 2.1 Plant material

A total of 480 fruit from the Japanese plum cultivars Santa Rosa and Sweet Miriam were harvested from a commercial orchard located in the California Central Valley (Parlier, CA, USA) during two seasons as previously described (Farcuh et al., 2017). Fruit growth and development patterns were monitored weekly and fruit were harvested at the ‘well-mature’ stage, corresponding to a flesh fruit firmness of ∼ 37 N (Farcuh et al., 2018). Fruit with uniform size, absence of visual blemishes, bruises and/or diseases were chosen.

### 2.2 Fruit postharvest storage and treatments

Fruit within each cultivar were randomized and assigned to one of two groups of 240 fruit each and commercially packed into cardboard boxes. Santa Rosa fruit from the first group were treated with 0.5 μL L^−1^ 1-MCP (SmartFresh^TM^, AgroFresh Inc., Spring House, PA, USA) at 20 °C for 24 h and immediately after treated were left to ripen under humidified, ethylene-free air at a flow rate of 2 L min^−1^ in 330-L sealed aluminum tanks connected to a flow-through system. Fruit from the second group, the controls, ripened under humidified, ethylene-free air at a flow rate of 2 L min^−1^ in 330-L sealed aluminum tanks connected to a flow-through system (Farcuh et al., 2018, Farcuh et al., 2019). Sweet Miriam fruit from the first group were left to ripen under humidified, ethylene-free air containing 500 μL L^-1^ of propylene (ethylene analogue, Praxair Inc., Danbury, CT, USA) at a flow rate of 2 L min^−1^ in 330-L sealed aluminum tanks connected to a flow-through system (Farcuh et al., 2018, Farcuh et al., 2019); while fruit from the second group, were treated as the Santa Rosa controls.

Fruit from all groups were stored at 20 °C and 90% relative humidity and evaluations were carried out at harvest (0) and after 1, 5, and 10d of storage (Supplementary Fig. S2). Santa Rosa fruit reached the “ready-to-eat” stage (≤10 N) and could not be further evaluated after 5d of postharvest. For each evaluation period, four biological replications from each group were assessed. For each biological replication, 6 fruit were used for assessing ethylene production rates and skin and flesh color, while 4 fruit were washed, peeled (skin tissue), cut into small pieces (flesh tissue). Each tissue type was pooled together, frozen and homogenized in liquid nitrogen, and stored at – 80 °C for further analyses.

### 2.3 Fruit ethylene production rates and color measurements

Fruit ethylene production rate (μL C_2_H_4_ kg^-1^h^−1^) and fruit skin and flesh color (hue angle) were measured as previously described (Farcuh et al., 2017, Kim et al., 2015).

### 2.4 Skin and flesh flavonoids quantification

#### 2.4.1 Plum skin and flesh flavonoid extraction

Skin and flesh tissue samples (1.0+/-0.2 g) were transferred to a 15 mL polypropylene test tube and 5.0 mL of extraction solvent (water:methanol (1:1 by volume) acidified with hydrochloric acid (0.1% of final volume containing ascorbic acid as antioxidant (1 g/L)) was added and the sample was immediately vortexed. Samples were extracted at 4 °C for 18 h with occasional agitation. Afterwards the samples were centrifuged (5 min at 3500 rpm, 4 °C) to pellet tissue and 1 mL of supernatant was sampled and clarified by centrifugation (5 min at 15,000 rpm, 4 °C) prior to reverse phase- high performance liquid chromatography (RP- HPLC) analysis.

#### 2.4.2 RP- HPLC analysis of plum skin and flesh flavonoid content

To determine the flavonoid content of plum skin and flesh tissue samples, RP-HPLC was performed. This would have not been possible with other detection methods, such as LC-MS, where differences in structure would have led to large errors in quantitation. RP-HPLC was used over spectroscopic methods, as it allows for differences of individual flavonoids to be determined rather than a bulk measurement done at a specific wavelength. RP-HPLC was conducted using a previously described method (Girardello et al., 2019) using an Agilent 1260 Infinity equipped with a PLRP-S 100A 3 μM 150 × 4.6 mm column (Agilent Technologies, Santa Clara, CA, USA) at 35 °C, an autosampler with temperature control at 8 °C, and diode array detector. Two mobile phases were used: mobile phase A (water containing 1.5% phosphoric acid v/v) and the mobile phase B (80% acetonitrile and 20% mobile phase A). Twenty μL of sample was injected with the mobile phase flow rate set at 1 mL/min. Instrument control and data analysis was performed in Agilent ChemStation ver. B.04.03. Flavonoids were detected at 280 nm ((+)-catechin, (-)-epicatechin, procyanidin B1, procyanidin B2), 360 nm (all flavonols), and 520 nm (all anthocyanins). The identity of flavonoids was confirmed by comparison of UV–VIS spectra to authentic standards when possible or to literature. Calibration curves were generated for (+)-catechin (used for all flavan-3-ols), isoquercitrin (quercetin-3-glucoside) (used for all flavonols), and cyanidin-3-glucoside (used for all anthocyanins). The concentration range for all calibration curves was 0.01 – 200 mg/100 g. Limit of quantitation was determined as the concentration giving a S/N of 10 and limit of detection as the concentration giving a S/N of 7 (Supplementary Table S1).

### 2.5 Real-time quantitative RT-PCR analysis

RNA was isolated as previously described (Kim, Saha, Farcuh, Li, Sadka, & Blumwald, 2015). First-strand complementary DNA synthesis, primer design, and quantitative PCR were performed as described before (Kim et al., 2015). The sets of primers used for the amplification of the different target genes are listed in Supplementary Table S2. The target genes analyzed in this study were selected based on two conditions: (i) their differential expression between fruit of Santa Rosa and Sweet Miriam cultivars and throughout developmental stages, using the RNASeq dataset and methodology for identification of differentially expressed genes, as described previously (Farcuh et al., 2017), and (ii) were previously reported in literature as key genes associated to the phenylpropanoid and flavonoid biosynthetic pathways. Analysis of the relative gene expression was performed according to the Comparative Cycle Threshold Method (Livak & Schmittgen, 2001). The expression of the SAND protein-related trafficking protein (*MON*) was used as a reference (Kim et al., 2015).

### 2.6 Statistical analyses

Means of the four biological replications for the assessed variables within each cultivar were submitted to two-way analysis of variance using posthoc Tukey’s test to compare between treatments and time in storage for significant differences (p ≤ 0.05).

Principal component analysis (PCA) was performed to analyze and visualize the relationships among all the analyzed flavonoid and sugar metabolism-related genes and contents. Pearson’s correlation coefficients (r), at a significance level ofα = 0.05, using log transformed and mean-centered data, were calculated for each pairwise-combination. Relative gene expression values for sugar metabolism-related genes, values for sugar contents, and relative gene expression values for ethylene metabolism were obtained from our prior studies (Farcuh et al., 2018, Farcuh et al., 2019). PCA produced a ‘biplot’ graph, representing the relationships among the analyzed variables and the evaluated plum cultivars. Scree test was used to determine the number of principal components required to capture most of the relevant variation in the data. Software package JMP (ver 14.0, SAS Institute, Cary, NC, USA) was used for analyses.

## 3 Results and discussion

### 3.1 Evaluation of ethylene production rates and plum skin and flesh coloration

The ripening behavior of fleshy fruits has been categorized as climacteric or non-climacteric, characterized by an upsurge in respiration rates and autocatalytic ethylene production in the former but not in the latter (Biale, Young, & Young, 1981). In this study, climacteric Santa Rosa control fruit displayed significantly increased ethylene production rates during ripening, while 1-MCP-treated fruits displayed dramatically lower ethylene production rates (Table 1). The reduction in ethylene production in 1-MCP-treated plum fruit is a result of the reduced transcription of ethylene biosynthesis-related mRNAs (Farcuh et al., 2019) as well as from the reduced expression of ethylene perception and signaling-associated genes (Cheng et al., 2016, Farcuh et al., 2019). Control and propylene-treated Sweet Miriam fruit maintained constant and low ethylene production rates during ripening (Table 1), with no induction of ethylene biosynthetic-related genes (Farcuh et al., 2019), supporting its non-climacteric nature.

**Table 1:** Fruit ethylene production rates and skin and flesh color of Santa Rosa (SR) and Sweet Miriam (SM) Japanese plum cultivars during ripening throughout postharvest storage.

		**Santa Rosa**							**Sweet Miriam**							
		Harvest		1 day at 20 °C		5 days at 20 °C			Harvest		1 day at 20 °C		5 days at 20 °C		10 days at 20 °C	
		Contr	1MCP	Contr	1MCP	Contr	1MCP		Contr	Prop	Contr	Prop	Contr	Prop	Cont	Prop
Ethylene production rate (µL C_2_H_4_ kg ^-1^h^−1^)		0.3 ± 0.03 d	0.3 ± 0.03 d	0.9 ± 0.3b	0.2 ± 0.02 d	46.7 ± 3.9 a	0.5 ± 0.08c		0.1 ± 0.02 a	0.1 ± 0.02 a	0.2 ± 0.01 a	0.1 ± 0.02 a	0.2 ± 0.04 a	0.2 ± 0.03 a	0.1 ± 0.01 a	0.1 ± 0.02 a
Fruit skin color (hue angle)		17.1 ±1.3 a	17.1 ± 1.3 a	16.7 ±1.1 a	17.5 ±1.4 a	11.6 ±0.4b	15.7 ±1.4 ab		47.6 ±3.3 a	47.6 ± 3.3 a	49.1 ± 3.7 a	36.9 ±2.9b	49.7 ± 4.4 a	33.0 ±1.7 bc	44.4 ± 3.7 a	25.2 ±0.6c
Fruit flesh color (hue angle)		70.6 ±2.6 a	70.6 ±2.6 a	65.5 ± 4.4 ab	70.0 ± 3.0 a	34.2 ± 1.6c	58.1 ± 3.2b		80.3 ± 0.6 a	80.3 ± 0.6 a	79.4 ± 0.7 a	78.4 ± 0.6 a	78.6 ± 0.4 a	76.6 ± 0.5 ab	78.1 ± 0.4 a	73.9 ± 0.3b

Values are means ± Standard Error (n = 4). Different letters indicate significant differences (p ≤ 0.05) according to Tukey’s test and are comparing within each cultivar/tissue. Control (Contr), 1- methylcyclopropane (1MCP), Propylene (Prop).

Japanese plum skin and flesh color are one of the most important determinants of fruit quality and marketability (Fanning et al., 2014). Skin and flesh hue values dramatically decreased in control and propylene-treated Santa Rosa and Sweet Miriam plums, respectively, throughout postharvest (Table 1). The decrease in hue values indicated an increase in red coloration, associated with the accumulation of fruit anthocyanins. Upon 1-MCP treatment, Santa Rosa fruit displayed significantly higher skin and flesh hue values as compared to control fruit, in agreement with the observed delay in color evolution in Japanese plums when ethylene perception was blocked (Pan, Wang, Li, Wang, Cao, & Jiang, 2016). Sweet Miriam control fruit skin and flesh hue values remained constant during ripening, and were significantly higher than propylene-treated fruits after 10 days of storage (Table 1). These results are consistent with studies suggesting that anthocyanin synthesis in plums is affected by ethylene (Cheng et al., 2016, Xu et al., 2020).

### 3.2 Effect of ethylene on key phenylpropanoid and flavonoid pathway-related genes and transcription factors associated with their regulation

#### 3.2.1 Anthocyanin biosynthetic genes

**Fig. 1:**
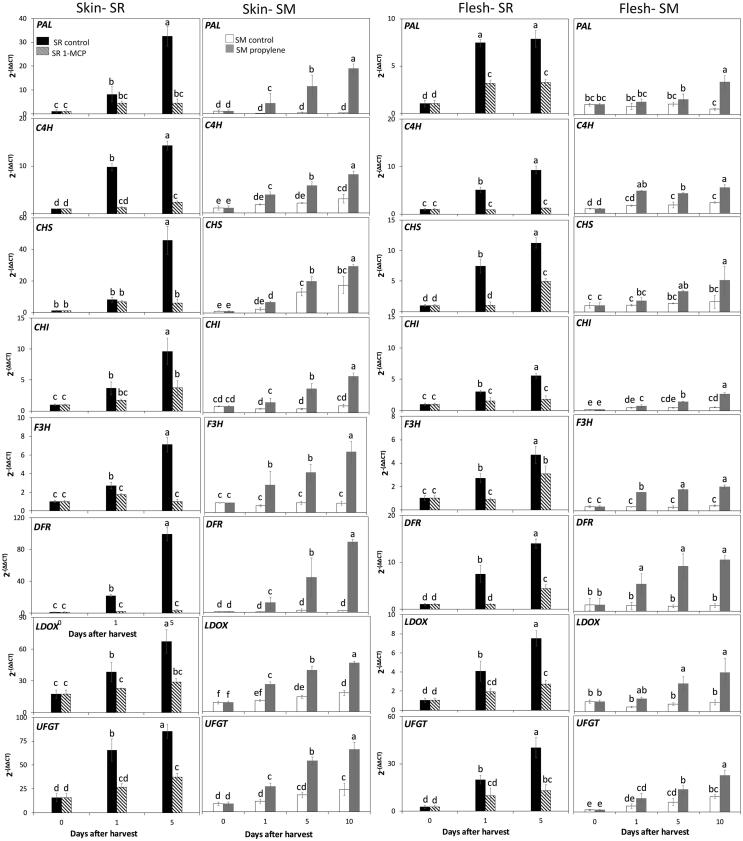
Relative gene expression levels of anthocyanin biosynthetic genes in skin and flesh tissues of Santa Rosa (SR) and Sweet Miriam (SM) plum cultivars submitted to ethylene treatments during ripening in storage. Values are means ± Standard Error (n = 4). Different letters indicate significant differences (p ≤ 0.05) according to Tukey’s test and are comparing within each cultivar/tissue. Phenylalanine ammonia-lyase (PAL), cinnamate-4-hydroxylase (C4H), chalcone synthase (CHS), chalcone isomerase (CHI), flavanone 3-hydroxylase (F3H), dihydroflavonol 4-reductase (DFR), leucoanthocyanidin dioxygenase (LDOX), UDP glucose-flavonoid 3-O-glucosyltransferase (UFGT), 1-methylcyclopropane (1-MCP).

To determine the role of ethylene regulation on anthocyanin biosynthesis, the expression profiles of eight key structural genes involved in anthocyanin biosynthesis, including *PAL, C4H, CHS, CHI, F3H, DFR, LDOX* and *UFGT*, were assessed in skin and flesh of Santa Rosa and Sweet Miriam cultivars subjected to different ethylene treatments (Fig. 1). Santa Rosa control fruit displayed a significant increase in expression of all anthocyanin-related genes in skin and flesh tissues throughout postharvest, which can be associated with increased ethylene production of this climacteric cultivar during storage (Table 1). Similar results were reported in skin tissue of other climacteric plum cultivars (Cheng et al., 2016, Xu et al., 2020), while continuous ethylene exposure in a climacteric plum led to a higher extractable PAL activity in flesh (Manganaris et al., 2008). In contrast, following 1-MCP treatment, Santa Rosa fruit showed lower transcript accumulation in all genes with respect to control fruit, with increase in expression only after 5 days of storage, in both tissues (Fig. 1), consistent with previous studies in other cultivars (Cheng et al., 2016, Xu et al., 2020).

Sweet Miriam fruit treated with propylene had significantly elevated levels of anthocyanin biosynthesis-related transcripts, with respect to control fruit, and displayed an increased expression during the ripening period in skin and flesh (Fig. 1). In agreement with our results, exogenous ethylene has also been reported to trigger gene expression of anthocyanin-related structural genes in other non-climacteric fruit such as grapes (Chervin et al., 2009, El-Kereamy et al., 2003) and strawberries (Villarreal et al., 2010). Thus, the results suggest that ethylene regulates anthocyanin biosynthesis-related genes in different tissues of climacteric and non-climacteric plums.

To further understand the relationship between ethylene metabolism and anthocyanin biosynthesis-related genes, correlation coefficients were calculated (Supplementary Table S3). For this we used the relative expression levels of key genes associated with ethylene biosynthesis (S-adenosyl-L-methionine synthase (*SAMS3),* 1- aminocyclopropane-1-carboxylic acid synthase *(ACS1, ACS3),* 1- aminocyclopropane-1-carboxylic acid oxidase *(ACO1, ACO3*)), ethylene perception (ethylene-response sensor (*ERS1),* ethylene receptor-types (*ETR1, ETR2),* ethylene insensitive (*EIN4)*), and ethylene signal transduction (ethylene-insensitive-like (*EIN3/EIL),* ethylene response factors *(ERFIX-6, ERFIX-7, ERFVII-6, ERFVIII-1*)) as previously reported (Farcuh et al., 2019) for flesh tissues of Santa Rosa and Sweet Miriam cultivars treated with ethylene during storage. Ethylene production rates were positively correlated with the transcription levels of all eight structural anthocyanin biosynthesis genes (r ≥ 0.64) (Supplementary Table S3). Furthermore, we observed positive correlations between the expression of ethylene and anthocyanin biosynthesis-related genes (r ≥ 0.58), further supporting the ethylene regulation of anthocyanin contents in plums. Transcription of genes encoding for ethylene receptors, which perceive ethylene and have an ethylene-binding capacity (Farcuh et al., 2019), were positively correlated with all anthocyanin biosynthesis-related genes (r ≥ 0.59). These results are supported by previous studies that reported a positive association between *ERS1* and *ETR1* with anthocyanin structural genes in plum (Cheng et al., 2016) and pear fruit (MacLean et al., 2007), suggesting their involvement in the anthocyanin biosynthetic pathway. Furthermore, positive correlations were obtained between expression levels of most ethylene signal transduction genes and all assayed anthocyanin-associated genes (r ≥ 0.61). The exception was *PsERFIX-6* which showed a negative correlation with *PAL, CHS, CHI, F3H*, and *UFGT* (r ≤ − 0.60)). This result is consistent with studies in plums indicating that ethylene is not the only regulator of the ethylene response factor *ERFIX-6* (Cheng et al., 2016, Farcuh et al., 2019), suggesting that *ERFIX-6* might not be involved in ethylene-regulated plum anthocyanin biosynthesis.

#### 3.2.2 Flavonol and flavan-3-ols biosynthetic genes

**Fig. 2:**
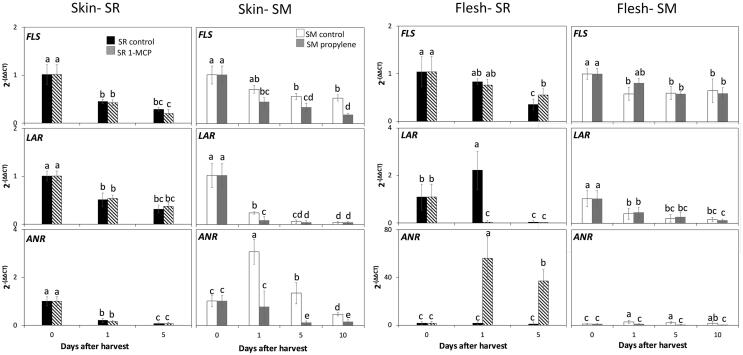
Relative gene expression levels of flavonol and flavan-3-ol biosynthetic genes in skin and flesh tissues of Santa Rosa (SR) and Sweet Miriam (SM) plum cultivars submitted to ethylene treatments during ripening in storage. Values are means ± Standard Error (n = 4). Different letters indicate significant differences (p ≤ 0.05) according to Tukey’s test and are comparing within each cultivar/tissue. Flavonol synthase (FLS), leucoanthocyanidin reductase (LAR), anthocyanidin reductase (ANR), 1-methylcyclopropane (1-MCP).

Anthocyanins, flavonol and flavan-3-ols are flavonoids and share common precursors of the phenylpropanoid and flavonoid pathways (Jaakola, 2013) (Supplementary Fig. S1). Transcript accumulation of *FLS*, encoding the key enzyme responsible for flavonol synthesis, and *LAR* and *ANR*, key genes in the synthesis of flavan-3-ols, were assessed to characterize the effect of ethylene on their regulation (Fig. 2). We did not observe consistent results indicating the induction of expression of *FLS, LAR, ANR* by ethylene throughout storage. On the contrary, Sweet Miriam control skin and flesh tissues as well as Santa Rosa 1-MCP-treated fruit flesh displayed an increased expression of *ANR* as compared to Sweet Miriam propylene-treated fruit and Santa Rosa control fruit respectively, suggesting a decrease in transcript accumulation due to ethylene. Furthermore, both cultivars displayed a decreased expression of all three assayed genes in skin and flesh tissues during postharvest (Fig. 2), contrasting to what was observed for anthocyanin biosynthesis-related genes (Fig. 1). These results would suggest a competition for substrates in the different branches of the phenylpropanoid and flavonoid pathways, which could be partly regulated by ethylene. Particularly, it has been reported that there is a competitive relation between DFR (directed towards anthocyanin biosynthesis) and FLS (directed towards flavonol synthesis) enzymes (Jaakola, 2013, Zhang et al., 2018), as well as between *UFGT* (directed towards anthocyanin biosynthesis) and *ANR* (directed towards flavan-3-ols synthesis) genes (Griesser et al., 2008). As the presence of ethylene increased the accumulation of *UFGT* transcripts (Fig. 1) (which is the limiting step in anthocyanin biosynthesis in fruit (Li, Zhang, Einhorn, & Cheng, 2014)), pathway intermediates would be targeted towards the promotion of anthocyanin biosynthesis, instead of flavonol and flavan-3-ol synthesis; while the opposite happened when ethylene was inhibited, as observed with the expression of *ANR* (Fig. 2). These results are further supported by the existence of significantly negative correlations (r ≤ − 0.50) (Supplementary Table S3) observed between some ethylene metabolism-associated gene expression levels (Farcuh et al., 2019) and *FLS*, *LAR* and *ANR* transcript accumulation.

#### 3.2.3 Transcription factors associated with the regulation of phenylpropanoid and flavonoid pathways

**Fig. 3:**
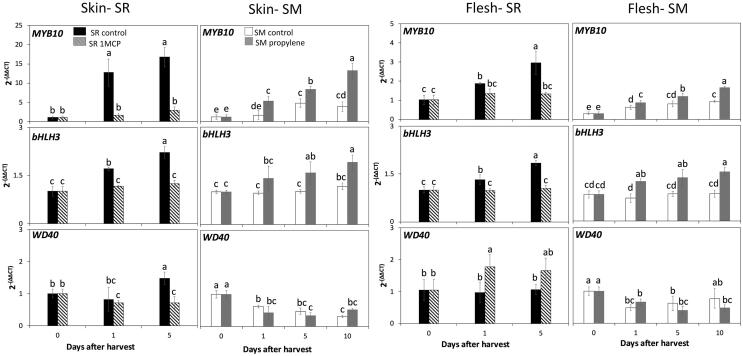
Relative gene expression levels of transcription factors associated with the regulation of phenylpropanoid and flavonoid pathways in skin and flesh tissues of Santa Rosa (SR) and Sweet Miriam (SM) plum cultivars submitted to ethylene treatments during ripening in storage. Values are means ± Standard Error (n = 4). Different letters indicate significant differences (p ≤ 0.05) according to Tukey’s test and are comparing within each cultivar/tissue. 1-methylcyclopropane (1-MCP).

Expression patterns of *MYB10*, *bHLH3* and *WD40*, members of the MBW complex, were assessed to determine their potential regulation of phenylpropanoid and flavonoid pathways and the role of ethylene in their expression in different tissues of both cultivars. Transcript accumulation of *MYB10* and *bHLH3* followed the same expression pattern and displayed positive correlations (r ≥ 0.74) with the eight assayed structural anthocyanin biosynthesis genes (Fig. 3, Supplementary Table S3). In contrast, an opposite expression pattern was observed between *MYB10* and *bHLH3* with flavonol biosynthesis gene *FLS*, with negative correlations (r ≤ − 0.56) while no significant correlations were detected with *LAR, ANR* (Supplementary Table S3). Our results suggest that these transcription factors play key roles as upstream regulators of anthocyanin biosynthesis in plum fruit. Accordingly, in Rosaceae species the expression levels of *MYB10* have been shown to induce anthocyanin biosynthesis pathways when co-expressed with bHLH3 by transactivating the DFR promoter (Lin-Wang et al., 2010), and in nectarines, *MYB10* positively regulated the promoter sequences of *DFR* and *UFGT*, but not *LAR* genes (Ravaglia et al., 2013). Particularly in plums, transcript accumulation of *MYB10* in climacteric ‘Oishi-wase’ plum was positively correlated with seven structural genes involved in anthocyanin biosynthesis (Cheng et al., 2016), while constitutive activation of *MYB10* in ‘Ziyeli’ plum was reported to be responsible for red pigmentation in the leaf, sepal, and fruit skin and flesh (Gu, Liao, Zhou, Wang, Deng, & Han, 2015).

Furthermore, the expression levels of *MYB10* and *bHLH3* positively correlated with ethylene production rates (r ≥ 0.77) (Supplementary Table S3), as their transcript accumulation increased during ripening of the Santa Rosa control and propylene-treated Sweet Miriam fruit, but was downregulated in 1-MCP-treated Santa Rosa and in Sweet Miriam control fruit (Fig. 3). These results are consistent with previous studies, showing that 1-MCP treatments decreased *MYB10* expression in climacteric plums (Cheng et al., 2016, Xu et al., 2020).

Correlation coefficients between the transcription levels of *MYB10* and *bHLH3* and key ethylene biosynthesis, perception and signaling-related genes (Farcuh et al., 2019) were significantly positive (r ≥ 0.65) (Supplementary Table S3). *WD40* expression did not follow the same trend as *MYB10* and *bHLH3*, and did not show significant correlations with flavonoid nor ethylene metabolism, indicating that although it is part of the MBW complex, it is not directly upregulated by ethylene. In apple, ethylene signaling-related genes (i.e., ethylene response factors (ERFs)) have been shown to bind to promoters of MYBs to alter anthocyanin biosynthesis (Zhang et al., 2018), while in pear, ERFs interacted with MYBs and bHLH3 to co-regulate anthocyanin biosynthesis (Yao et al., 2017). The results suggest that the effect of ethylene on anthocyanin biosynthesis in climacteric and non-climacteric plums during postharvest may be via the regulation of *MYB10* and *bHLH3* expression by their interaction with ERFs, particularly *ERFIX-7, ERFVII-6,* and *ERFVIII-1,* acting as positive regulators of ethylene-induced responses (Supplementary Table S3). In a previous study we showed that these ERFs are well conserved in both ripening types and involved in inducing ethylene-related physiological responses (Farcuh et al., 2019). Positive correlations between expression profiles of ERFs with *MYB10* and anthocyanin biosynthesis structural genes were also reported in climacteric ‘Oishi-wase’ plum (Cheng et al., 2016).

### 3.3 Analysis of flavonoid concentration

#### 3.3.1 Anthocyanin concentration

Three anthocyanins were detected in this study, including cyanidin-3-glucoside (Cy3Glu), cyanidin-3-rutinoside (Cy3Rut), and cyanidin-3-galactoside (Cy3Gal) (Table 2), with the latter reported as a characteristic anthocyanin of Santa Rosa plums (Tomás-Barberán et al., 2001). Cy3Glu was the predominant anthocyanin in both skin and flesh tissues of both plum cultivars, in agreement with previous studies (Xu et al., 2020, Yu et al., 2021). Higher contents for all anthocyanins were detected in skin as compared to flesh tissues, consistent with the higher hue values displayed by the latter (Table 1) and previous reports (Tomás-Barberán et al., 2001).

**Table 2:** Concentrations of anthocyanins, flavonols and flavan-3-ols (mg 100 g^−1^ FW) in skin and flesh tissues of Santa Rosa and Sweet Miriam Japanese plum cultivars submitted to different ethylene treatments during ripening in postharvest storage.

	**Santa Rosa**						**Sweet Miriam**							
Flavonoids/Tissue	Harvest		1 day at 20 °C		5 days at 20 °C		Harvest		1 day at 20 °C		5 days at 20 °C		10 days at 20 °C	
	Contr	1MCP	Contr	1MCP	Contr	1MCP	Contr	Prop	Contr	Prop	Contr	Prop	Cont	Prop
**Anthocyanins**														
Cy3Glu- Skin	102.3 ± 5.7c	102.3 ± 5.7c	210.3 ± 23.2b	102.82 ± 13.5c	309.1 ± 40 a	126.9 ± 23.8 bc	4.2 ± 0.2c	4.2 ± 0.2c	4.9 ± 0.1c	9.9 ± 1.1b	4.6 ± 0.2c	13.4 ± 1.4b	7.6 ± 0.3 bc	37.6 ± 2.6 a
Cy3Glu- Flesh	1.1 ± 0.1 d	1.1 ± 0.1 d	7.9 ± 0.3b	2.7 ± 0.3 cd	12.5 ± 0.5 a	5.2 ± 0.3 bc	0.2 ± 0.05 d	0.2 ± 0.05 d	0.4 ± 0.04 d	1.5 ± 0.09c	0.5 ± 0.05 d	3.0 ± 0.1b	1.9 ± 0.2c	5.6 ± 0.1 a
Cy3Rut- Skin	23.7 ± 1.4 d	23.7 ± 1.4 d	77.4 ± 7.2 ab	44.2 ± 5.5 cd	97.9 ± 7.6 a	53.8 ± 10.2 bc	1.1 ± 0.03 d	1.1 ± 0.03 d	2.4 ± 0.09 cd	5.7 ± 0.08b	2.6 ± 0.1 cd	5.1 ± 0.08b	3.1 ± 0.07c	9.9 ± 0.06 a
Cy3Rut- Flesh	0.5 ± 0.05 d	0.5 ± 0.05 d	1.2 ± 0.09b	0.4 ± 0.1 d	1.6 ± 0.1 a	0.8 ± 0.1c	0.07 ± 0.01 d	0.07 ± 0.01 d	0.08 ± 0.01 d	0.2 ± 0.05 cd	0.1 ± 0.02 d	0.7 ± 0.1b	0.5 ± 0.1c	1.1 ± 0.1 a
Cy3Gal- Skin	16 ± 0.9c	16 ± 0.9c	32.9 ± 3.5b	16.8 ± 2.5c	49 ± 6.2 a	20.4 ± 3.7 bc	0.9 ± 0.03c	0.9 ± 0.03c	0.5 ± 0.01c	2 ± 0.04b	0.9 ± 0.04c	2.7 ± 0.04b	2.4 ± 0.06b	7.5 ± 0.06 a
Cy3Gal- Flesh	0.2 ± 0.02 d	0.2 ± 0.02 d	1.5 ± 0.2b	0.2 ± 0.04 d	2.2 ± 0.1 a	0.8 ± 0.1c	0.1 ± 0.01 d	0.1 ± 0.01 d	0.2 ± 0.04 d	0.4 ± 0.04 bc	0.2 ± 0.03 cd	1 ± 0.1b	0.7 ± 0.04c	1.5 ± 0.1 a
**Flavonols**														
Isoquer- Skin	20.4 ± 2.3b	20.4 ± 2.3b	18.3 ± 3.1b	45.8 ± 3.2 a	9.5 ± 1.3c	42.7 ± 2.9 a	50.4 ± 3.3b	50.4 ± 3.3b	55.1 ± 2b	27.6 ± 1.5c	69.9 ± 3.7 ab	15.3 ± 1.3 d	80.6 ± 3.1 a	14.1 ± 1.2 d
Isoquer- Flesh	0.4 ± 0.1b	0.4 ± 0.1b	0.2 ± 0.01c	0.7 ± 0.1 a	0.2 ± 0.03c	0.8 ± 0.1 a	0.7 ± 0.04b	0.7 ± 0.04b	0.9 ± 0.04b	0.4 ± 0.03c	1.2 ± 0.1 a	0.4 ± 0.04c	1.4 ± 0.04 a	0.4 ± 0.04c
Quer- Skin	9.3 ± 0.4b	9.3 ± 0.4b	6.2 ± 0.5c	12.7 ± 0.9 a	5.7 ± 0.4c	13.5 ± 0.7 a	13 ± 0.6 ab	13 ± 0.6 ab	15.9 ± 0.6 a	10.3 ± 1b	13.6 ± 0.8 ab	3.8 ± 0.8c	16.9 ± 0.5 a	4.6 ± 0.3c
Rutin- Skin	41.6 ± 2.4b	41.6 ± 2.4b	27.1 ± 1.8c	57.2 ± 6.3 a	21.5 ±. 8c	62.5 ± 2.6 a	56.8 ± 2.2b	56.8 ± 2.2b	95.9 ± 4.3 a	41.9 ± 7.3 bc	85.1 ± 6.5 ab	25.7 ± 3.7c	116.8 ± 2.2 a	27.5 ± 2.6c
Avicularin- Skin	3.5 ± 0.1b	3.5 ± 0.1b	2 ± 0.1c	3.9 ± 0.2 ab	2.3 ± 0.1c	4.8 ± 0.2 a	47.6 ± 3.3b	47.6 ± 3.3b	49.1 ± 3.7 a	36.9 ± 2.9b	49.7 ± 4.4b	33 ± 1.7c	44.4 ± 3.7 a	25.2 ± 0.6c
**Flavan-3-ols**														
Catechin- Skin	13.4 ± 0.7b	13.4 ± 0.7b	15.8 ± 0.5b	35.5 ± 0.7 a	12.5 ± 0.7b	40.2 ± 0.8 a	21.7 ± 0.6b	21.7 ± 0.6b	36.2 ± 0.9 a	19.5 ± 0.7b	39.8 ± 0.9 a	16.8 ± 0.5 bc	42.3 ± 0.8 a	15.2 ± 0.6c
Catechin- Flesh	15.1 ± 0.6c	15.1 ± 0.6c	10.2 ± 0.6 d	25.1 ± 0.8b	9.4 ± 0.4 d	35.8 ± 0.9 a	20.9 ± 0.3c	20.9 ± 0.3c	28.7 ± 0.3b	16.7 ± 0.6 cd	35.7 ± 1 ab	10.4 ± 0.9 d	39.3 ± 0.3 a	16.3 ± 0.6 cd
Epicatechin- Skin	7.2 ± 0.5c	7.2 ± 0.5c	6.9 ± 0.8c	12.8 ± 0.2b	6.6 ± 0.8c	17.3 ± 0.7 a	12.9 ± 0.4b	12.9 ± 0.4b	22.3 ± 0.6 a	11.5 ± 0.6b	22.6 ± 0.5 a	9.2 ± 0.5 bc	25.1 ± 0.8 a	8.9 ± 0.5c
Epicatechin- Flesh	9.8 ± 0.3b	9.8 ± 0.3b	7.5 ± 0.4c	14.4 ± 0.4 a	6.4 ± 0.3c	13.3 ± 0.2 a	14.5 ± 0.4b	14.5 ± 0.4b	18.2 ± 0.3 ab	11.5 ± 0.9 bc	20.3 ± 0.8 a	10.3 ± 0.7c	21.7 ± 0.5 a	6.9 ± 0.6 d
ProcyB1- Skin	11.5 ± 1.2b	11.5 ± 1.2b	13.7 ± 1.7b	30.5 ± 0.4 a	11.4 ± 1.7b	33.5 ± 1 a	19.8 ± 1.3b	19.8 ± 1.3b	23.4 ± 1b	10 ± 0.9c	40 ± 0.7 a	8.4 ± 0.6c	45.1 ± 0.6 a	10.3 ± 0.7c
ProcyB1- Flesh	9.9 ± 0.4b	9.9 ± 0.4b	8.9 ± 0.5b	25.8 ± 0.8 a	4.3 ± 0.4c	28.2 ± 0.6 a	17.6 ± 0.8b	17.6 ± 0.8b	29.1 ± 1.4 ab	8.3 ± 0.7c	28.9 ± 0.5 ab	7.7 ± 0.6c	35.8 ± 0.9 a	10.9 ± 0.5c
ProcyB2- Skin	10.4 ± 0.3b	10.4 ± 0.3b	9.1 ± 1.4b	14.9 ± 1.2 a	4.5 ± 0.4c	16.4 ± 0.5 a	13.5 ± 0.3b	13.5 ± 0.3b	19.8 ± 0.4 a	12.9 ± 0.3b	20.2 ± 0.2 a	7.8 ± 0.5c	22.6 ± 0.2 a	8.2 ± 0.5c
ProcyB2- Flesh	9.9 ± 0.4b	9.9 ± 0.4b	8.9 ± 0.6b	25.8 ± 0.8 a	4.3 ± 0.4c	28.2 ± 0.6 a	17.6 ± 0.8b	17.6 ± 0.8b	29.1 ± 1.4 ab	8.3 ± 0.7c	28.9 ± 0.5 ab	7.7 ± 0.7c	35.8 ± 0.9 a	10.9 ± 0.5c

Values are means ± Standard Error (n = 4). Different letters indicate significant differences (p ≤ 0.05) according to Tukey’s test and are comparing within each cultivar/tissue. Cyanidin-3-glucoside (Cy3Glu), Cyanidin-3-rutinoside (Cy3Rut), Cyanidin-3-galactoside (Cy3Gal), Isoquercitrin (Isoquer), Quercitrin (Quer), Procyanidin B1(ProcyB1), Procyanidin B2 (ProcyB2), Control (Contr), 1- methylcyclopropane (1MCP), Propylene (Prop).

In response to ethylene, a significant increase in contents of all three detected anthocyanins in both tissues of Santa Rosa control fruit throughout postharvest was observed. However, 1-MCP treatment induced a two to three-fold decrease in anthocyanin contents as compared to control fruit (Table 2). In Sweet Miriam, propylene application significantly increased all three anthocyanins by two-to six-fold with respect to control fruit in skin and flesh tissues (Table 2). These results agree with the significant differences in transcript accumulation of anthocyanin biosynthesis- related genes and associated transcription factors (Fig. 1, 3), as well as with differences in skin and flesh coloration between control and treated fruit in each cultivar (Table 1). Consistently, application of 1-MCP in climacteric plums was reported to significantly decrease skin anthocyanin contents throughout storage with respect to untreated fruit (Xu et al., 2020), while exogenous ethylene application increased anthocyanin accumulation in plum fruit skin (Cheng et al., 2016) and flesh (Manganaris et al., 2008). Furthermore, exogenous ethylene treatment was also shown to increase anthocyanin contents in non-climacteric grapes (El-Kereamy et al., 2003) and strawberries (Villarreal et al., 2010). The results further support the involvement of ethylene in the regulation of anthocyanin contents in both ripening types.

#### 3.3.2 Flavonol and Flavan-3-ol concentration

Four flavonols, including rutin (quercetin-3-rutinoside), isoquercitrin (quercetin-3-glucoside), quercitrin (quercetin-3-rhamnoside), and avicularin (quercetin-3-arabinoside); and four flavan-3-ols, including catechins, epicatechins, procyanidin B1 and B2, were detected in this study (Table 2), in agreement with previous reports in Japanese plums (Fanning et al., 2014, Jaiswal et al., 2013, Venter et al., 2013, Yu et al., 2021). All flavonols were detected in skin tissues, while in flesh only isoquercitrin was observed in both cultivars, and in significantly lower contents as compared to skin (Table 2). Flavonols have been reported to be usually most abundant in fruit skin and to decrease towards the flesh (Tomás-Barberán et al., 2001). All four flavan-3-ols were detected in both skin and flesh tissues in both cultivars, with similar contents, consistent with results in other *Rosaceae* fruit (Treutter, 2001).

Among skin flavonols, rutin was the most abundant compound together with isoquercitrin in both cultivars, as reported previously (Tomás-Barberán et al., 2001, Venter et al., 2013). In the flavan-3-ol category, catechin and procyanidin B1 were the most abundant compounds in both tissues of Santa Rosa and Sweet Miriam cultivars, consistent with previous studies (Jaiswal et al., 2013, Venter et al., 2013).

In response to ethylene, and opposing to what was observed for anthocyanin contents, the results showed constant or significantly lower contents of all detected flavonols and flavan-3-ols in skin and flesh tissues of Santa Rosa control fruit throughout postharvest (Table 2). However, upon 1-MCP treatment flavonols and flavan-3-ols contents increased up to four-fold in both tissues as compared to control fruit (Table 2). In Sweet Miriam, propylene application decreased flavonols and flavan-3-ols contents by two to five-fold with respect to control fruit in skin and flesh tissues (Table 2). These results are in agreement with the significantly higher expression of *ANR* genes in 1-MCP treated Santa Rosa and control Sweet Miriam fruits with respect to Santa Rosa control or propylene-treated Sweet Miriam, respectively (Fig. 2) and are consistent with the notion that when anthocyanin biosynthesis is decreased, there are more phenylpropanoid and flavonoid pathways intermediates available for the synthesis of flavonols and flavan-3-ols due to substrate competition. In strawberries, UFGT-silenced fruit was shown to redirect anthocyanin biosynthesis into flavan-3-ol production (Griesser et al., 2008), while silencing the expression of anthocyanidin synthase gene in a red-leaved apple cultivar blocked anthocyanin synthesis and increased flavonols and flavan-3-ols accumulation in leaves (Szankowski et al., 2009). The lack of increase in transcript accumulation of *FLS* and *LAR* after 1-MCP application in Santa Rosa and in Sweet Miriam control fruit (Fig. 2), in contrast to what was observed for their associated metabolites (Table 2) would suggest possible posttranscriptional regulation of the transcripts and/or posttranslational modifications of the enzyme activities coded by these genes, and needs to be explored further.

### 3.4 Relationships among ethylene production rates and key phenylpropanoid, flavonoid and sugar metabolism-related genes and contents

**Fig. 4:**
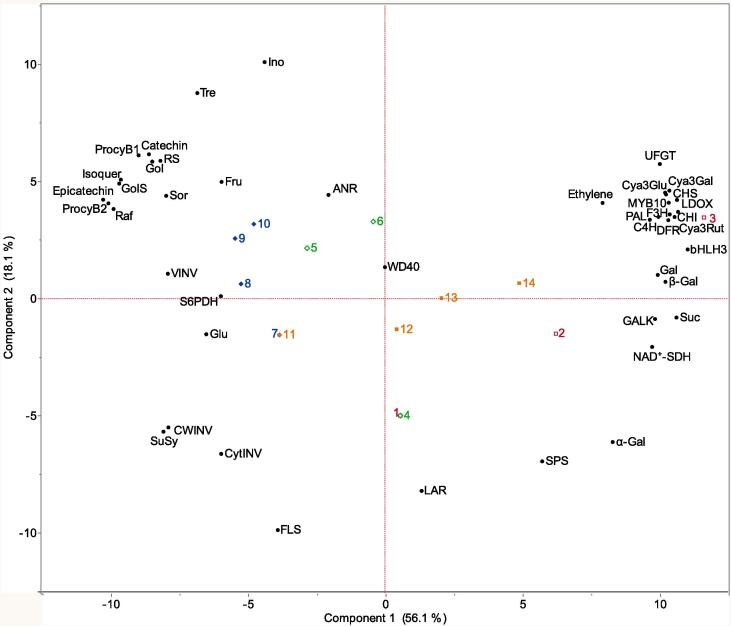
Biplot from Principal Component Analysis of ethylene production rates, key phenylpropanopid, flavonoid and sugar metabolism-related genes and contents of flesh tissue of Santa Rosa (SR) and Sweet Miriam (SM) plum cultivars during ripening in postharvest storage. Numbers correspond to different cultivar/postharvest evaluation period/ ethylene treatment #1 (SR/Harvest/control), #2 (SR/1 day at 20 °C/control), #3 (SR/5 days at 20 °C/control), #4 (SR/Harvest/1-MCP), #5 (SR/1 day at 20 °C/1-MCP), #6 (SR/5 days at 20 °C/1-MCP), #7 (SM/Harvest/control), #8 (SM/1 day at 20 °C/control), #9 (SM/5 days at 20 °C/control), #10 (SM/10 days at 20 °C/control), #11 (SM/Harvest/propylene), #12 (SM/1 day at 20 °C/ propylene), #13 (SM/5 days at 20 °C/ propylene), #14 (SM/10 days at 20 °C/ propylene). Codes for genes and metabolite contents correspond to codes in Fig. 1, Fig. 2, Fig. 3, Table 2 and (Farcuh et al., 2018).

Sugars, in addition to ethylene, have been reported to regulate phenylpropanoid and flavonoid pathways (Duran-Soria et al., 2020). Previously (Farcuh et al., 2018), we conducted gene expression profiling of 11 key sugar metabolism-related genes associated with the major sugars sucrose (Suc), sorbitol (Sor), fructose (Fru) and glucose (Glu), and the minor sugars galactose (Gal), galactinol (Gol), raffinose (Raf), myo-inositol (Ino), and trehalose (Tre) in flesh tissue of Santa Rosa and Sweet Miriam plums exposed to ethylene treatments, demonstrating a reprogramming of sugar metabolism in both cultivars regulated by ethylene. In this study, correlation coefficients were calculated and a Principal Component Analysis (PCA) was performed (Fig. 4) to visualize the relationships among changes in ethylene production rates and key phenylpropanoid, flavonoid and sugar metabolism-related genes and contents in flesh tissue of both plum cultivars.

The PCA showed that the first and second principal components explained 56.1% (Component 1) and 18.1% (Component 2) of the observed variation (74.2% total). On the first principal component, the separation of each cultivar/postharvest evaluation period/ethylene treatment combination was driven by the contents of the flavan-3-ols epicatechin and procyanidin B2 on the negative side (associated with 1-MCP-treated Santa Rosa and control Sweet Miriam fruit) and by Suc contents and transcript accumulation of *bHLH3* on the positive side (associated with control Santa Rosa and propylene-treated Sweet Miriam fruit) (Fig. 4). The distribution of each cultivar/postharvest evaluation period/ethylene treatment combination along Component 1 of the PCA is supported by differences in ethylene, as Santa Rosa control and propylene-treated Sweet Miriam fruit were constantly exposed to high ethylene rates, while 1-MCP-treated Santa Rosa and control Sweet Miriam fruit had reduced ethylene rates (Table 1) as well as expression of ethylene metabolism-related genes (Farcuh et al., 2019).

Analyzed variables that presented statistically higher values in control Santa Rosa and propylene-treated Sweet Miriam fruit were located on the positive (right) side of the biplot (Fig. 1, Fig. 2, Fig. 3, Fig. 4, Table 1, Table 2) (Farcuh et al., 2018). Contents of the three detected anthocyanins and expression profiles of all assayed anthocyanin-related structural and regulatory genes were positively correlated with Suc and Gal contents (r ≥ 0.68) as well as with Suc (sucrose phosphate synthase (*SPS*)) and Gal (beta-galactosidase (BGAL), alpha-galactosidase (AGAL), and galactokinase (GALK)) synthesis-related genes (r ≥ 0.51), while negatively correlated with Suc breakdown-related genes, particularly invertases (cell wall invertase (*CWINV),* cytosolic invertase (*CytINV));* (r ≤ − 0.59)) and Suc synthase (*SuSy;* (r ≤ − 0.57) (Fig. 4). While the positive correlations exhibited between anthocyanins and Suc have been reported previously in grapes (Zheng et al., 2009), apricots (Huang et al., 2019) and peaches (Zhou et al., 2020), this is the first report in plums. Consistently, in vitro transcriptomic analysis of grape berries demonstrated that anthocyanin content enhancement by Suc results from important expression changes of both regulatory and structural genes of the phenylpropanoid and flavonoid pathways (Dai et al., 2013). For Gal, the positive correlation with anthocyanin-related genes and contents, could be supported by Gal being a major sugar donor for anthocyanin production, and additionally by the possible synergistic interaction between ethylene and free Gal, that could promote anthocyanin biosynthesis. Free Gal has been reported to stimulate ethylene production and promote earlier ripening in tomatoes due to the capacity of Gal to promote the activity of ACS, the rate-limiting step in ethylene biosynthesis (Kim, Gross, & Solomos, 1987), although this is currently under investigation.

In contrast to anthocyanins, flavonol and flavan-3-ol contents as well as transcript accumulation of *FLS* and *ANR*, were negatively correlated with Suc and Gal contents and their biosynthesis-related genes (r ≤ − 0.60), as well as to the Sor breakdown-related gene Sor dehydrogenase (*NAD^+^-SDH;* r ≤ − 0.55); while positively correlated to transcript accumulation of Suc breakdown-related genes such as vacuolar invertase (*VINV*; r ≥ 0.70), contents of Sor (r ≥ 0.60), Fru (r ≥ 0.59), Glu (r ≥ 0.50) as well as minor sugars such as Raf (r ≥ 0.68), Gol (r ≥ 0.58), Ino (r ≥ 0.53) and Tre (r ≥ 0.54) including their biosynthesis-related genes (Fig. 4). Consistently, the highest flavonol and flavan-3-ol contents were associated with the 1-MCP-treated Santa Rosa and Sweet Miriam control plums during ripening (Fig. 2, Table 2), supporting their positioning in the PCA (Fig. 4). These results further supported the existence of a competition for substrates in the different branches of the phenylpropanoid and flavonoid pathways, as reported previously in strawberry (Griesser et al., 2008) and apples (Szankowski et al., 2009). The results would suggest that in the presence of ethylene, Suc or Gal the substrates in the phenylpropanoid and flavonoid pathways might mainly flow towards anthocyanin synthesis. On the other hand, under low ethylene production rates, and high contents of Sor, Fru, Glu or minor sugars, anthocyanin synthesis is suppressed, and the biosynthetic pathways are redirected towards flavonol and flavan-3-ol accumulation. Consistently, in terms of Sor metabolism, a study in grape suspension cells showed that neither mannitol nor Sor were able to enhance anthocyanin contents (Larronde, Krisa, Decendit, Cheze, Deffieux, & Mérillon, 1998), while in red-blushed apricots Sor was negatively correlated with anthocyanin skin contents (Huang et al., 2019). In pears, the enhanced anthocyanin synthesis in the skin of the bud mutant red ‘Anjou’ led to upregulation of Sor breakdown through NAD^+^-SDH in both skin and flesh tissues (Li et al., 2014). Furthermore, and in agreement with our results, 1-MCP-treated ‘Taoxingli’ plums maintained higher contents of Sor, Glu and Fru, and lower Suc contents compared to control fruit, as well as a remarkably suppressed expression of several anthocyanin structural synthesis-related genes and anthocyanin contents during postharvest (Xu et al., 2020). In terms of minor sugars, our results in plum flesh differ with a study in the skin of the pear bud mutant red ‘Anjou’ where an increase in raffinose synthase activity and in Raf and Ino contents were associated with increased anthocyanin (Li et al., 2014). These differences need further investigation and could be explained by the occurrence of a sugar-specific regulation of phenylpropanoid and flavonoid pathways in different fruit tissues or even fruit species.

## 4 Conclusions

Gene expression and metabolite evaluation of phenylpropanoid and flavonoid-related pathways in skin and flesh tissues of Santa Rosa and its non-climacteric mutant Sweet Miriam during postharvest, integrated with multivariate analyses of ethylene and sugar metabolism, demonstrated that changes in ethylene and sugar-related pathways are important factors affecting the overall regulation of flavonoid metabolism. Ethylene shows contrasting effects on anthocyanin versus flavonol and flavan-3-ol metabolism: it induces transcription of anthocyanin biosynthesis structural and regulatory genes and anthocyanin contents, but tends to decrease flavonol and flavan-3-ol contents in skin and flesh tissue of both cultivars. Ethylene biosynthesis, and ethylene perception and signaling-related genes correlate positively with anthocyanin biosynthesis structural and regulatory genes, but display an antagonistic or no relation with flavonol and flavan-3-ol-related genes. Furthermore, Suc and Gal contents and synthesis-related genes positively correlate with anthocyanin contents and transcript accumulation of structural and regulatory genes, while Sor, Fru, Glu and minor sugars and their biosynthesis-related genes are positively associated with flavonol and flavan-3-ol contents and related genes.

Our results support the notion that anthocyanin biosynthesis competes for substrates with flavonols and flavan-3-ols, and further suggest that ethylene and sugars play a key role(s) in regulating these pathways and shifting fruit flavonoid profiles of climacteric and non-climacteric plums during postharvest. This work could be applied to the identification and manipulation of potential targets for improvement of plum fruit coloration and health properties.

## Declaration of Competing Interest

The authors declare that they have no known competing financial interests or personal relationships that could have appeared to influence the work reported in this paper.
