# Supplementary material for: Changes in ethylene and sugar metabolism regulate flavonoid composition in climacteric and non-climacteric plums during postharvest storage
Source: Food Chem (Oxf). 2022 Jan 21;4:100075. doi: 10.1016/j.fochms.2022.100075 (PMC8991838; doi:10.1016/j.fochms.2022.100075)
Supplement: Supplementary data 1 [file mmc1.docx]

**Supplementary Table S1.** Wavelength, retention time, LOD and LOQ values, and calibration equations and curves used for quantitation of flavonoids in plum skin and flesh tissues by RP-HPLC.

| **Flavonoids** | **Flavonoid Class** | **Retention time (min.)** | **Wavelength (nm)** | **LOQ (mg/L)*** | **LOD (mg/L)**** | **Calibration equation** | **R^2^** | **Calibration Curve used for quantitation** | |  |
| --- | --- | --- | --- | --- | --- | --- | --- | --- | --- | --- |
| (+)-Catechin | Flavan-3-ol | 26.3 | 280 | 0.5 | 0.2 | y=44.031x+30.653 | 0.999 | (+)-Catechin | |  |
| (-)-Epicatechin | Flavan-3-ol | 30.2 | 280 | 0.5 | 0.2 | y=44.031x+30.653 | 0.999 | (+)-Catechin | |  |
| Procyanidin B1 | Flavan-3-ol | 26.9 | 280 | 0.5 | 0.2 | y=44.031x+30.653 | 0.999 | (+)-Catechin | |  |
| Procyanidin B2 | Flavan-3-ol | 36.0 | 280 | 0.5 | 0.2 | y=44.031x+30.653 | 0.999 | (+)-Catechin | |  |
| Rutin (Quercetin-3-rutinoside) | Flavonol | 57.1 | 360 | 0.2 | 0.1 | y=21.161x+2.6015 | 0.9998 | Isoquercitrin (Quercetin-3-glucoside) | |  |
| Isoquercitrin (Quercetin-3-glucoside) | Flavonol | 49.4 | 360 | 0.2 | 0.1 | y=21.161x+2.6015 | 0.9998 | Isoquercitrin (Quercetin-3-glucoside) | |  |
| Quercitrin (Quercetin-3-rhamnoside) | Flavonol | 59.6 | 360 | 0.2 | 0.1 | y=21.161x+2.6015 | 0.9998 | Isoquercitrin (Quercetin-3-glucoside) | |  |
| Avicularin (Quercetin-3-arabinoside) | Flavonol | 55.3 | 360 | 0.2 | 0.1 | y=21.161x+2.6015 | 0.9998 | Isoquercitrin (Quercetin-3-glucoside) | |  |
| Cyanidin-3-glucoside | Anthocyanin | 25.1 | 520 | 0.3 | 0.2 | y=22.998x+10.236 | 0.9998 | Cyanidin-3-glucoside | |  |
| Cyanidin-3-galactoside | Anthocyanin | 23.3 | 520 | 0.3 | 0.2 | y=22.998x+10.236 | 0.9998 | Cyanidin-3-glucoside | |  |
| Cyanidin-3-rutinoside | Anthocyanin | 27.6 | 520 | 0.3 | 0.2 | y=22.998x+10.236 | 0.9998 | Cyanidin-3-glucoside | |  |
| *LOQ determined as the calibration concentration giving a signal-to-noise of approximately 10 | | | | | | | | |  | |
| **LOD determined as the calibration concentration giving a signal-to-noise of approximately 7 | | | | | | | | | | |
