# Supplementary material for: Changes in ethylene and sugar metabolism regulate flavonoid composition in climacteric and non-climacteric plums during postharvest storage
Source: Food Chem (Oxf). 2022 Jan 21;4:100075. doi: 10.1016/j.fochms.2022.100075 (PMC8991838; doi:10.1016/j.fochms.2022.100075)
Supplement: Supplementary data 2 [file mmc2.docx]

**Supplementary Table S2.** Primers used in qRT-PCR

| **Gene name** | **Prunus persica gene ID** | **Description** | **Primer orientation** | **Primer sequence**  **(5’ to 3’)** |
| --- | --- | --- | --- | --- |
| PAL | ppa002099m.g | Phenylalanine ammonia-lyase | Forward | CTATCTGCGAGGGAAAGATTATC |
|  |  |  | Reverse | GGACACAGAAGTAGTGGAATGG |
| DFR | ppa008069m.g | Dihydroflavonol 4-reductase | Forward | CGAAGAGCACCAGAAGTCATAC |
|  |  |  | Reverse | GTCGAAGTACATCCAACCAGTC |
| CHS | ppa008402m.g | Chalcone synthase | Forward | GAATTTGATGCGTCTGCTTCCTTG |
|  |  |  | Reverse | CATTGCCCACACACGTTTCCAAC |
| C4H | ppa004544m.g | Cinnamate acid 4-monooxygenase | Forward | GGGATCACTTTGGGACGTTTAG |
|  |  |  | Reverse | CACCTTTCTCTGTGGTGTCAAG |
| CHI | ppa011276m.g | Chalcone isomerase | Forward | ACCCAGGTGTACAAGAGAAATG |
|  |  |  | Reverse | GAGCACTTTCGTAGCTTCATAGA |
| F3H | ppa007636m.g | Flavanone 3-hydroxylase | Forward | GGGCAAGCCAAAGGATGATA |
|  |  |  | Reverse | GAACTAGACGGCAAAGGTACAA |
| FLS | ppa008322m.g | Flavonol synthase | Forward | CATGTCCATCGTCACCATTCT |
|  |  |  | Reverse | TGTGGATGACAAGGGCATTAG |
| LDOX | ppa007738m.g | Leucoanthocyanidin dioxygenase | Forward | TGGGTTGGGATTGGAAGAAG |
|  |  |  | Reverse | GGGCAAAGTGGGTAGTAGTT |
| LAR | ppa007994m.g | Leucoanthocyanidin reductase | Forward | CCCTACTTCGACAACAAGCA |
|  |  |  | Reverse | CCAATATCAGCGCCGTCTATAA |
| ANR | ppa008244m.g | Anthocyanidin reductase | Forward | TTCCATGTAGCAACTCCCTTAC |
|  |  |  | Reverse | ACCACAGAAGCTGTGTAGATTAG |
| UFGT | ppa005162m.g | UDP glucose-flavonoid 3- o -glucosyl transferase | Forward | ATTACCGACGCCTTCCTTTG |
|  |  |  | Reverse | CATGCAGGGAGAGGGAATTAAG |
| MYB10 | ppa026640m.g | Transcription factor | Forward | AGTGGCACCAAGTTCCTAAC |
|  |  |  | Reverse | CTCTGCAAACTCTCCTCTCTTG |
| BHLH3 | ppa002884m.g | Transcription factor | Forward | CTCCAATGCCCGTATAGAGAAG |
|  |  |  | Reverse | CTCAGCTACGAAGAACCCATTAT |
| WD40 | ppa008187m.g | Transcription factor | Forward | GATATCCGTTCGCCGACTATG |
|  |  |  | Reverse | GCAGAGCAGATGTGTCTACAA |
| MON | ppa003026m.g | SAND-related trafficking protein | Forward | GGGAACCTTATATTGGCGTAGG |
|  |  |  | Reverse | CTGCTGACGAGGACTACTTATTG |
