# Supplementary material for: Changes in ethylene and sugar metabolism regulate flavonoid composition in climacteric and non-climacteric plums during postharvest storage
Source: Food Chem (Oxf). 2022 Jan 21;4:100075. doi: 10.1016/j.fochms.2022.100075 (PMC8991838; doi:10.1016/j.fochms.2022.100075)
Supplement: Supplementary data 3 [file mmc3.docx]

**Supplementary Table S3.** Pearson correlation coefficients among ethylene production rates, and the expression levels of phenylpropanoid, flavonoid and ethylene metabolism-related genes and transcription factors in flesh tissue of Santa Rosa (SR) and Sweet Miriam (SM) Japanese plum cultivars during ripening in postharvest storage at 20ºC.

|  | *PAL* | *C4H* | *CHS* | *CHI* | *F3H* | *DFR* | *LDOX* | *UFGT* | *FLS* | *LAR* | *ANR* | *MYB10* | *bHLH3* | *WD40* |
| --- | --- | --- | --- | --- | --- | --- | --- | --- | --- | --- | --- | --- | --- | --- |
| Ethylene | 0.69* | 0.74* | 0.69* | 0.81* | 0.72* | 0.64* | 0.79* | 0.79* | -0.58* | NS | NS | 0.86* | 0.77* | NS |
| **Ethylene biosynthesis** | | | | | | | | | | | | | | |
| *SAMS3* | 0.67* | 0.85* | 0.66* | 0.77* | 0.72* | 0.84* | 0.75* | 0.75* | NS | NS | -0.55* | 0.76* | 0.89* | NS |
| *ACS1* | 0.66* | 0.76* | 0.72* | 0.85* | 0.76* | 0.65* | 0.83* | 0.81* | -0.52* | NS | NS | 0.80* | 0.69* | NS |
| *ACS3* | 0.80* | 0.65* | 0.81* | 0.89* | 0.83* | 0.58* | 0.82* | 0.77* | -0.50* | NS | NS | 0.86* | 0.65* | NS |
| *ACO1* | 0.83* | 0.67* | 0.87* | 0.91* | 0.87* | 0.63* | 0.86* | 0.81* | -0.52* | NS | NS | 0.88* | 0.68* | NS |
| *ACO3* | 0.72* | 0.73* | 0.80* | 0.87* | 0.81* | 0.66* | 0.84* | 0.83* | -0.52* | NS | NS | 0.82* | 0.68* | NS |
| **Ethylene perception** | | | | | | | | | | | | | | |
| *ERS1* | 0.90* | 0.63* | 0.90* | 0.89* | 0.84* | 0.68* | 0.84* | 0.80* | -0.50* | NS | NS | 0.88* | 0.65* | NS |
| *ETR1* | 0.73* | 0.68* | 0.63* | 0.70* | 0.66* | 0.65* | 0.63* | 0.66* | -0.52* | NS | NS | 0.75* | 0.65* | NS |
| *ETR2* | 0.65* | 0.84* | 0.69* | 0.67* | 0.59* | 0.85* | 0.63* | 0.66* | NS | NS | -0.55* | 0.65* | 0.83* | NS |
| *EIN4* | 0.80* | 0.77* | 0.73* | 0.84* | 0.77* | 0.69* | 0.80* | 0.76* | NS | NS | -0.52* | 0.81* | 0.71* | NS |
| **Ethylene signaling** | | | | | | | | | | | | | | |
| *EIN3/EIL* | 0.77* | 0.87* | 0.75* | 0.85* | 0.85* | 0.85* | 0.85* | 0.81* | NS | NS | -0.52* | 0.83* | 0.86* | NS |
| *ERFIX-6* | -0.70* | NS | -0.60* | -0.60* | -0.70* | NS | NS | -0.60* | NS | NS | NS | -0.71* | -0.70* | NS |
| *ERFIX-7* | 0.72* | 0.84* | 0.77* | 0.90* | 0.85* | 0.77* | 0.89* | 0.85* | -0.56* | NS | NS | 0.83* | 0.80* | NS |
| *ERFVII-6* | 0.61* | 0.80* | 0.69* | 0.66* | 0.71* | 0.73* | 0.66* | 0.66* | -0.52* | NS | NS | 0.67* | 0.74* | NS |
| *ERFVIII-1* | 0.63* | 0.83* | 0.68* | 0.79* | 0.76* | 0.77* | 0.78* | 0.73* | -0.52* | NS | -0.58* | 0.75* | 0.80* | NS |
| **Flavonoid- related transcription factors** | | | | | | | | | | | | | | |
| *MYB10* | 0.85* | 0.81* | 0.90* | 0.98* | 0.90* | 0.80* | 0.94* | 0.95* | -0.60* | NS | NS | 1.00* | 0.87* | NS |
| *bHLH3* | 0.75* | 0.93* | 0.74* | 0.87* | 0.84* | 0.94* | 0.90* | 0.92* | -0.56* | NS | NS | 0.87* | 1.00* | NS |
| *WD40* | NS | NS | NS | NS | NS | NS | NS | NS | NS | NS | NS | NS | NS | 1.00* |

All correlations shown are significant (*; P≤0.05) except NS (non-significant).
