# Supplementary material for: Changes in ethylene and sugar metabolism regulate flavonoid composition in climacteric and non-climacteric plums during postharvest storage
Source: Food Chem (Oxf). 2022 Jan 21;4:100075. doi: 10.1016/j.fochms.2022.100075 (PMC8991838; doi:10.1016/j.fochms.2022.100075)
Supplement: Supplementary data 4 [file mmc4.pptx]

## Slide 1
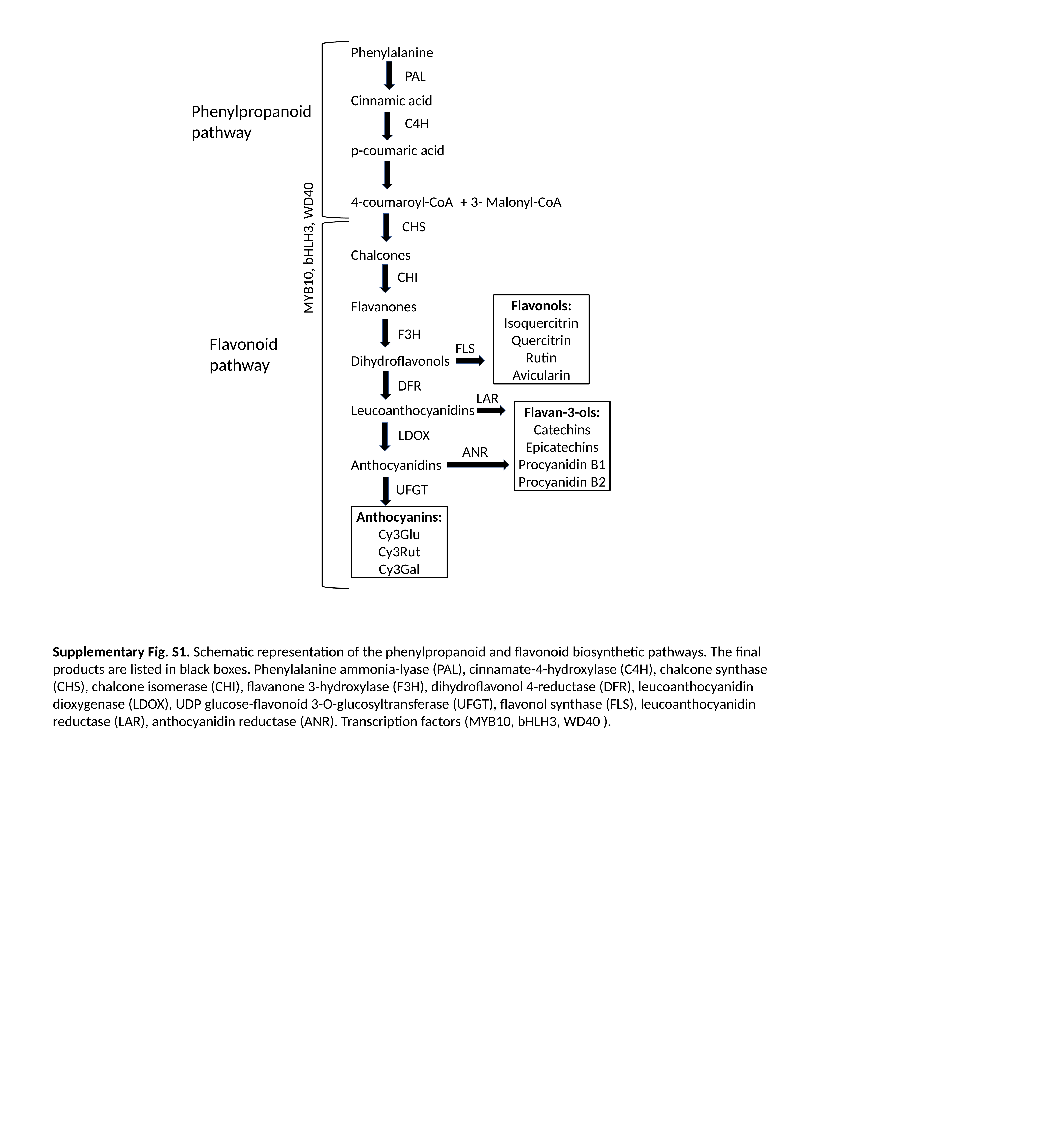

Phenylalanine
PAL
Cinnamic acid
Phenylpropanoid pathway
C4H
p-coumaric acid
+ 3- Malonyl-CoA
4-coumaroyl-CoA
CHS
MYB10, bHLH3, WD40
Chalcones
CHI
Flavonols:
Isoquercitrin
Quercitrin
Rutin
Avicularin
Flavanones
F3H
Flavonoid
pathway
FLS
Dihydroflavonols
DFR
LAR
Leucoanthocyanidins
Flavan-3-ols:
Catechins
Epicatechins
Procyanidin B1
Procyanidin B2
LDOX
ANR
Anthocyanidins
UFGT
Anthocyanins:
Cy3Glu
Cy3Rut
Cy3Gal
Supplementary Fig. S1. Schematic representation of the phenylpropanoid and flavonoid biosynthetic pathways. The final products are listed in black boxes. Phenylalanine ammonia-lyase (PAL), cinnamate-4-hydroxylase (C4H), chalcone synthase (CHS), chalcone isomerase (CHI), flavanone 3-hydroxylase (F3H), dihydroflavonol 4-reductase (DFR), leucoanthocyanidin dioxygenase (LDOX), UDP glucose-flavonoid 3-O-glucosyltransferase (UFGT), flavonol synthase (FLS), leucoanthocyanidin reductase (LAR), anthocyanidin reductase (ANR). Transcription factors (MYB10, bHLH3, WD40 ).
