# Supplementary material for: Changes in ethylene and sugar metabolism regulate flavonoid composition in climacteric and non-climacteric plums during postharvest storage
Source: Food Chem (Oxf). 2022 Jan 21;4:100075. doi: 10.1016/j.fochms.2022.100075 (PMC8991838; doi:10.1016/j.fochms.2022.100075)
Supplement: Supplementary data 5 [file mmc5.pptx]

## Slide 1
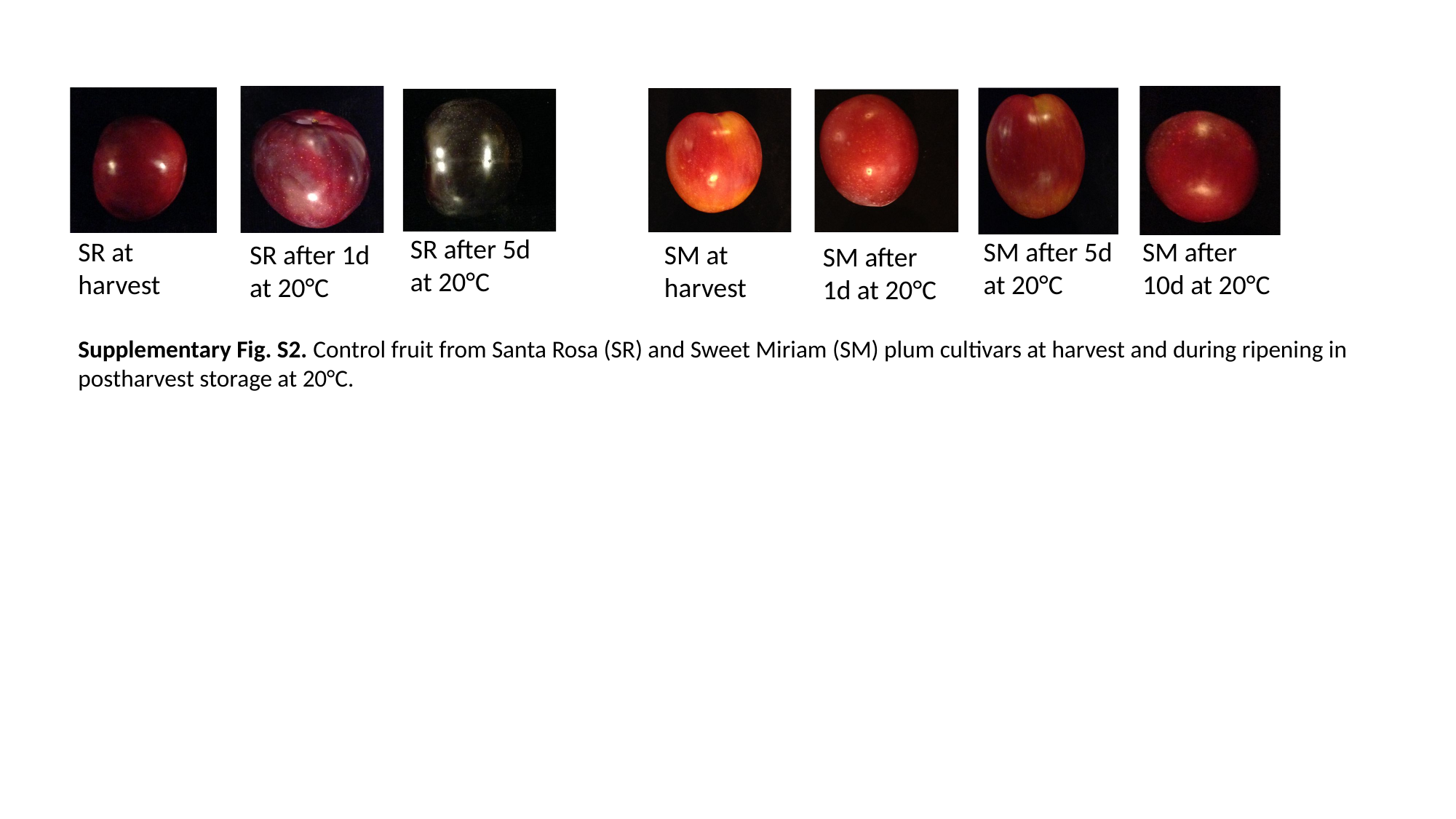

SR after 5d at 20°C
SR at harvest
SM after 5d at 20°C
SM after 10d at 20°C
SM at harvest
SR after 1d at 20°C
SM after 1d at 20°C
Supplementary Fig. S2. Control fruit from Santa Rosa (SR) and Sweet Miriam (SM) plum cultivars at harvest and during ripening in postharvest storage at 20°C.
